# Supplementary material for: Spoken language and attitudes in Hong Kong: English leads in prestige, mandarin rises in employability, and Cantonese faces challenges
Source: PLoS One. 2026 Mar 9;21(3):e0343611. doi: 10.1371/journal.pone.0343611 (PMC12970906; doi:10.1371/journal.pone.0343611)
Supplement: Attachment — Submitted filename: Response.docx [file pone.0343611.s003.docx]

Dear Dr. Berghoff and Reviewers,

Thank you very much for your careful consideration of our manuscript. We are sincerely grateful for the insightful comments and constructive suggestions from both the editorial team and each reviewer. Your feedback has been invaluable in strengthening our work.

In response to your major concerns, we have:

1. **Clarified our Methods.**
   We reorganized the Methods section into five clear subsections—Participants, Materials, Design, Procedure, and Analysis—added Cronbach’s α values for all composite scales, and provided a concise justification for using Welch’s ANOVA under relaxed homogeneity assumptions.
2. **Enhanced figure quality.**
   All placeholder images have been replaced with publication-ready, high-resolution figures, and we confirmed that every graphic is CC BY-compatible.
3. **Deepened theoretical grounding.**
   We integrated the key theories and models highlighted by the reviewers into our Introduction—such as Ethnolinguistic Vitality Theory and the Stereotype Content Model—to better frame our hypotheses and situate our findings.

We believe these revisions have substantially improved clarity, rigor, and overall impact. Thank you again for your time, effort, and thoughtful advice. We appreciate the opportunity to resubmit and look forward to your further guidance.

Sincerely,

Xiangbin Teng
Assistant Professor, Department of Psychology
The Chinese University of Hong Kong

Journal Requirements:

When submitting your revision, we need you to address these additional requirements.

1. Please ensure that your manuscript meets PLOS ONE's style requirements, including those for file naming. The PLOS ONE style templates can be found at

https://journals.plos.org/plosone/s/file?id=wjVg/PLOSOne_formatting_sample_main_body.pdf and

<https://journals.plos.org/plosone/s/file?id=ba62/PLOSOne_formatting_sample_title_authors_affiliations.pdf>

Reply: Thanks for the suggestion. We have gone through the requirements and revised our manuscript and submission accordingly

2. We note that the grant information you provided in the ‘Funding Information’ and ‘Financial Disclosure’ sections do not match.

When you resubmit, please ensure that you provide the correct grant numbers for the awards you received for your study in the ‘Funding Information’ section.

3. Thank you for stating the following financial disclosure:

“Improvement on Competitiveness in Hiring New Faculties Funding Scheme, the Chinese University of Hong Kong (4937113), to X.T.”

Please state what role the funders took in the study. If the funders had no role, please state: "The funders had no role in study design, data collection and analysis, decision to publish, or preparation of the manuscript."

If this statement is not correct you must amend it as needed.

Please include this amended Role of Funder statement in your cover letter; we will change the online submission form on your behalf.

Reply: Thank you for the comment, the funding information has been amended on lines 30-32

4. Thank you for uploading your study's underlying data set. Unfortunately, the repository you have noted in your Data Availability statement does not qualify as an acceptable data repository according to PLOS's standards.

At this time, please upload the minimal data set necessary to replicate your study's findings to a stable, public repository (such as figshare or Dryad) and provide us with the relevant URLs, DOIs, or accession numbers that may be used to access these data. For a list of recommended repositories and additional information on PLOS standards for data deposition, please see https://journals.plos.org/plosone/s/recommended-repositories.

Reply: Thank you for bringing this to our attention. The OSF link originally included in our submission was temporarily flagged due to an internal spam detection issue on the OSF platform. We have contacted OSF support, and the issue has now been resolved. The data repository is once again fully accessible to the public.

5. We note that Figure 1 in your submission contain copyrighted image. All PLOS content is published under the Creative Commons Attribution License (CC BY 4.0), which means that the manuscript, images, and Supporting Information files will be freely available online, and any third party is permitted to access, download, copy, distribute, and use these materials in any way, even commercially, with proper attribution. For more information, see our copyright guidelines: http://journals.plos.org/plosone/s/licenses-and-copyright.

We require you to either (1) present written permission from the copyright holder to publish these figures specifically under the CC BY 4.0 license, or (2) remove the figures from your submission:

a. You may seek permission from the original copyright holder of Figure 1 to publish the content specifically under the CC BY 4.0 license.

We recommend that you contact the original copyright holder with the Content Permission Form (http://journals.plos.org/plosone/s/file?id=7c09/content-permission-form.pdf) and the following text:

“I request permission for the open-access journal PLOS ONE to publish XXX under the Creative Commons Attribution License (CCAL) CC BY 4.0 (http://creativecommons.org/licenses/by/4.0/). Please be aware that this license allows unrestricted use and distribution, even commercially, by third parties. Please reply and provide explicit written permission to publish XXX under a CC BY license and complete the attached form.”

Please upload the completed Content Permission Form or other proof of granted permissions as an "Other" file with your submission.

In the figure caption of the copyrighted figure, please include the following text: “Reprinted from [ref] under a CC BY license, with permission from [name of publisher], original copyright [original copyright year].”

b. If you are unable to obtain permission from the original copyright holder to publish these figures under the CC BY 4.0 license or if the copyright holder’s requirements are incompatible with the CC BY 4.0 license, please either i) remove the figure or ii) supply a replacement figure that complies with the CC BY 4.0 license. Please check copyright information on all replacement figures and update the figure caption with source information. If applicable, please specify in the figure caption text when a figure is similar but not identical to the original image and is therefore for illustrative purposes only.

Reply: Thank you for the clarification. The elements in Figure 1 that were previously under copyright have been replaced with alternatives that are free for use under the Creative Commons Attribution (CC BY) license. We have also updated the figure caption to reflect appropriate attribution.

### Reviewer #1: A. Major Strengths

1. Timely and Contextually Rich

Addresses a pressing issue in Hong Kong’s evolving linguistic landscape post handover, protests, and pandemic lockdowns, adding empirical data to discussions of identity, power, and language prestige.

Reply: We sincerely thank the reviewer for recognizing the relevance and timeliness of our study.

2. Robust Sample and Design

• Large sample of N = 541 local participants after exclusions, with balanced gender distribution.

• Use of ten trilingual speakers and 67 neutral sentences reduces content and speaker confounds.

Reply: We appreciate the reviewer’s recognition of our efforts to ensure methodological rigor.

3. Rigorous Audio Processing

• Detailed denoising, filtering, and normalization procedures ensure stimulus quality and comparability across languages.

Reply: Thank you for noting this. We applied denoising, filtering, and normalization to ensure cross-language comparability of stimuli to ensure acoustic consistency

4. Comprehensive Trait Measures

• The combination of McCroskey’s Source Credibility items and employability questions captures both social cognitive and pragmatic attitudes.

Reply: We thank the reviewer for highlighting this point.

5. Statistical Treatment

• Appropriate use of Welch’s ANOVA, Tukey corrections, and reporting of effect sizes (means, SDs, F values, p values) enhances transparency.

Reply: We appreciate the reviewer’s recognition of our rigorous approach.

B. Major Weaknesses

1. Accent and Proficiency Control

Speakers are Hong Kong–born second language users of English and Mandarin. Regional accent may confound “language” effects with “accent” effects.

Please include objective measures of speaker proficiency and perceived accent strength. At minimum, discuss this limitation more explicitly and consider adding a post hoc listener rating of accent authenticity.

Reply: We thank the reviewer for highlighting this important point. We agree that the use of Hong Kong–born speakers introduces the possibility that accent familiarity may have influenced listeners’ evaluations independently of language. While we aimed to maintain ecological validity by using fluent local speakers, we acknowledge that this design choice may have confounded language with accent. Relevant changes have been made to the “Limitations” section on line 621-629 where we discuss the confounding influence of accent and propose future improvements.

We did not obtain objective measures of speaker proficiency or listener-rated accent strength, which limits our ability to isolate language effects from accent-based perceptions. We have now made this limitation more explicit in the “Limitations and Future Directions” section of the Discussion and noted how future studies could improve upon this by incorporating speaker proficiency benchmarks and accent norming procedures. Discussion of accent as a confound and implications of speaker proficiency has been expanded in the Discussion.

2. Warmth/Trust Measures Null Effects

The absent differences in warmth or trustworthiness across languages contradict both SCM predictions and some prior Cantonese-favoring studies. Maybe the authors should explore whether scale items lacked sensitivity (e.g., ceiling effects) or whether participants misunderstood definitions by reporting scale reliabilities (Cronbach’s α) for each dimension in those, which would clarify measure validity.

Reply: We thank the reviewer for raising this important point regarding potential measurement issues in the warmth/trustworthiness dimension. To assess the reliability of our trait measures, we calculated Cronbach’s α for the composite scales used in the analysis. Internal consistency was high across all dimensions: Trustworthiness (α = .87), Competence (α = .88), and Goodwill/Benevolence (α = .87). These results suggest that the trait scales were reliable and unlikely to have lacked sensitivity due to item inconsistency. We have now included these values in the manuscript to clarify scale validity, Cronbach’s α values are now reported in the Results section, on Lines 422–425.

3. Recruitment and Representativeness

I have always been cautious with convenience sampling (university emails, social media), because it may skew toward younger, more educated, or more English/Mandarin-proficient respondents, affecting the representativeness of the sample. Thus, it is very important to provide participant demographics beyond gender (e.g., age distribution, education level, self-rated language proficiency) to assess generalizability.

Reply: We appreciate the reviewer’s concern regarding sample representativeness and acknowledge the importance of reporting broader demographic details. Due to the anonymous nature of our survey and efforts to minimize participant burden, we did not collect data beyond gender. As such, we are unable to provide information on participants’ age, education level, or self-rated language proficiency in this dataset.

We recognize that this limits the generalizability of our findings and have now explicitly acknowledged this limitation in the Limitations and Future Directions section on lines 630-636. We also note that our recruitment strategy—while based on convenience sampling—is consistent with common practice in language attitudes research and yielded a large sample (N = 541) of self-identified Hong Kong locals, helping to mitigate some concerns about sampling bias. In future work, we plan to include a more detailed demographic profile to enhance interpretability and external validity.

4. Data Availability Statement

While data are said to be in an OSF folder, the exact DOI is provided “view only” and may not be accessible post publication. Please ensure a non-restricted data DOI is registered (non–“view only”) or deposit it in a recognized repository with persistent identifiers.

Reply: Thank you for bringing this to our attention. The OSF link originally included in our submission was temporarily flagged due to an internal spam detection issue on the OSF platform. We have contacted OSF support, and the issue has now been resolved. The data repository is once again fully accessible to the public.

5. Interpretation of Mandarin’s Favorability

The explanation that Hong Kong–accented Mandarin reduces outgroup bias is plausible but speculative without direct evidence. Please tone down conjecture or support with listener-rated accent familiarity/liking data. Alternatively, plan a follow-up study manipulating accent.

Reply: We appreciate the reviewer’s thoughtful observation regarding the explanation of Hong Kong–accented Mandarin reducing outgroup bias. We agree that our original interpretation may appear speculative without direct listener-rated data on accent familiarity or preference. Accordingly, we have revised the relevant paragraph in the Discussion section to clarify that this explanation remains a hypothesis and to explicitly acknowledge the lack of empirical evidence within the current study. We now frame this point more cautiously and suggest future research to investigate accent perception more directly, for instance through explicit manipulation or listener-rated accent strength on lines 572-583

C. Minor Comments

1. Literature Citations: Some DOI links are broken or incomplete in the reference list (e.g., Coupland & Bishop, 2007 has “doi/ 10.1111…” with a space). Please correct the formatting.

Reply: Thank you for pointing this out. We have reviewed the reference list and corrected formatting issues in the DOIs

2. Figure Quality: Figures 1 and 2 are informative but small in the PDF; ensure high-resolution versions for publication.

Reply: Thanks for pointing this out. The figures inserted are only for viewing. We uploaded figures with high resolution with the revised manuscript.

3. Typographical Errors:

Line 91: “use e provides” → “use provides”

Line 167: “administered using Qualtrics (Version 2023.5, Qualtrics, 2023. https://www.qualtrics.com).” Consider moving the URL to the reference list.

Reply: We thank the reviewer for their attentive reading and helpful suggestions. All minor editorial issues have been addressed.

Line 95: “e “ has be removed

Line 360: link has been moved to reference list

4. Ethics Statement: The text notes recruitment dates and consent but omits the institutional review board’s approval number in the Ethics Statement section.

Reply: Thank you for the comment. We would like to clarify that the approval number is included in the manuscript: “This study was approved by the Survey and Behavioural Research Ethics Committee of the Chinese University of Hong Kong (Study I.D. = dre20261).” We have reviewed the statement for clarity and will ensure it is appropriately formatted and visible in the final version.

In sum, the study addresses an important research question with a solid design and dataset. However, substantial clarifications and additional analyses (particularly regarding accent/proficiency controls, scale reliabilities, and participant demographics) are needed before the manuscript meets PLOS ONE’s standards for methodological rigor and interpretive strength.

Reply: We appreciate the reviewer’s thoughtful summary and their recognition of the study’s relevance and design. We have substantially revised the manuscript to address the concerns raised, including clarifying methodological details, strengthening the theoretical framing, and refining the interpretation of findings. We trust these revisions enhance the manuscript’s clarity, rigor, and contribution to the field.

### Reviewer #2:

This paper reports on a language attitudes study that investigates how Hong Kong locals evaluate local varieties of Cantonese, Mandarin and English. It does so building on the matched-guise paradigm. While this set-up is promising and could lead to valuable insights, the paper falls short in a number of ways. (1) The study is not sufficiently grounded in the (sociolinguistic) literature on language attitudes. (2) Further, and potentially as a result of the former, central concepts to the study are not rigorously defined and used throughout the article. For instance, the authors mention implicit attitudes a few times, but these are not relevant with regards to this specific study, as the MGT does not allow for the measurement of such attitudes. Another issue is the exact focus of the evaluation: it is not clear what aspects of social evaluation (which attributes or evaluative dimensions if you will) are targeted in the study which makes the argument sometimes hard to follow. (3) The text could benefit from a revision aimed at increasing its coherence. (4) The methods section is not logically structured and there are many unclarities regarding the methodology. (5) The analysis is not up to standards. As a result, I recommend major revisions. I hope the comments below, organized per section of the paper, will clarify the points above and will be helpful to the authors in their revision. I wish them the best of luck with their study.

Reply: We thank the reviewer for their thoughtful and comprehensive feedback, as well as for recognizing the potential of the study. We appreciate the detailed suggestions provided throughout the review, which have been instrumental in guiding our revisions. In the revised manuscript, we have made substantial changes to address each of the concerns raised, including: strengthening the theoretical grounding in sociolinguistic language attitudes literature, clarifying core concepts (e.g., our use of “implicit” evaluations), defining the evaluative traits and their connection to theoretical frameworks such as the Stereotype Content Model, and reorganizing the Methods section for clarity and transparency.

We hope that these revisions adequately address the concerns and improve the clarity, coherence, and contribution of the manuscript. Detailed point-by-point responses follow below.

>>Data availability

- The OSF link included in the submission is not publicly accessible. Hence, I have not been able to review any of the materials submitted with the article.

Reply: Thank you for bringing this to our attention. The OSF link originally included in our submission was temporarily flagged due to an internal spam detection issue on the OSF platform. We have contacted OSF support, and the issue has now been resolved. The data repository is once again fully accessible to the public.

Significance statement:

- The statement mentioned the study reveals implicit biases, however, the methodology employed (the MGT) does not allow for the measurement of attitudes under implicit conditions. Hence, this claim is incorrect.

Reply: We thank the reviewer for this important observation and acknowledge that the term “implicit attitudes” can carry different interpretations across disciplines. While our study does not employ implicit measures in the strict cognitive psychology sense, within sociolinguistics and social psychology, the matched-guise technique (MGT) is widely regarded as an indirect method for eliciting social judgments that participants may be unwilling or unable to report explicitly. By concealing the study’s true purpose, the MGT reduces social desirability bias, thereby functioning as an implicit measure in this disciplinary context.

Although participants provided explicit ratings, the experimental manipulation remained implicit because participants were unaware of the linguistic variation being evaluated. This indirectness underpins the strength of the MGT in revealing socially sensitive attitudes, such as language-based biases.

Importantly, our use of a between-subjects design—where each participant heard only one speaker in one language—further minimized the likelihood of detecting the manipulation, enhancing the covert nature of the attitude assessment. This methodological choice supports the interpretation that the MGT captures language-based social judgments that may operate outside conscious awareness, consistent with broader definitions of “implicit” in language attitude research.

In summary, while we concur that the MGT does not measure implicit attitudes in the cognitive psychology sense, it remains an implicit technique insofar as it indirectly reveals social evaluations by masking the linguistic manipulation. We have retained our original framing in the manuscript while clarifying this disciplinary distinction in our response on lines 187-192

>>Introduction

- “Such associations between speakers' traits and their languages reflect social dynamics and have implications for employment and government policies […]” (l.75-76): This remains quite vague. Please be more concrete and discuss examples based on the literature.

Reply: We thank the reviewer for pointing out the vagueness of this sentence. In the revised manuscript, we elaborated on how language-based social judgments can influence real-world outcomes. For example, previous studies have demonstrated that language can affect professional credibility (Evans, 2011). In Hong Kong, English is often associated with access to higher education and high-paying jobs, while Mandarin is increasingly required in government positions and sectors linked to mainland China (Lai, 2012; Choi, 2010). We have revised this sentence to reflect these concrete examples and their implications for employment and policy making in multilingual societies on lines 78-83

- The study is not well situated in the rich literature on language attitudes and the matched-guise tradition. Moreover, many statements and claims are not supported by references. One example is lines 93-97 which do not include a single reference while making quite broad claims.

Reply: We thank the reviewer for pointing this out. In the originally submitted version, we introduced general claims about the sociocultural functions of language without providing supporting references at that early point in the Introduction. While those claims were elaborated and supported with specific citations later in the same section, we acknowledge the importance of anchoring them from the beginning. We have revised the relevant sentences to include appropriate references (e.g., Lai, 2012; Newman et al., 2008; Coupland & Bishop, 2007), which are now on line 98-103 thereby situating the study more clearly within the literature on language attitudes and the matched-guise tradition.

- The study refers to the Stereotype Content Model as a theoretical underpinning of the association between language and social evaluation. However, no reference at all is made to theorizing on this matter in the field of sociolinguistics, the dominant field in the study of language attitudes and the social meaning of language. While I appreciate alternative theoretical approaches and do not suggest the authors should limit their theoretical perspective to the dominant theorizing in sociolinguistics, the existing sociolinguistic literature cannot be ignored as is currently the case in this article. Furthermore, the SCM seems to align well with findings from the long tradition of language attitudes research, but this is not explicitly discussed. The authors just cite three examples without a more thorough discussion of the field at large.

Reply: We sincerely appreciate the reviewer’s thoughtful feedback regarding the theoretical positioning of our study. We agree that the rich tradition of sociolinguistic research on language attitudes and social meaning—particularly within the matched-guise paradigm—offers essential context for understanding how listeners evaluate speakers across different linguistic varieties.

In the revised manuscript, we have substantially expanded the Introduction to better integrate this literature. We now explicitly reference key sociolinguistic theories, including Ethnolinguistic Vitality Theory (Giles, Bourhis, & Taylor, 1977), and draw connections between traditional constructs such as status, prestige, and solidarity and the Stereotype Content Model’s dimensions of competence and warmth. We have also incorporated additional foundational works (e.g., Coupland & Bishop, 2007; Garrett, 2010; Trenchs-Parera & Newman, 2009) to demonstrate that our use of SCM is not in opposition to sociolinguistic theorizing but rather complements it by offering a structured cognitive framework for trait-based evaluations.

We believe these revisions address the concern that SCM was previously presented in a disjointed or isolated manner, and we hope that this improved theoretical integration clarifies how our study contributes to both sociolinguistic and social cognitive perspectives on language attitudes. The additions are made on lines 104-114.

- In their discussion of competence stereotypes, the authors implicitly compare the status of Spanish in Catalonia with that of English in Hong Kong. I’m not entirely convinced of the parallel, so perhaps the authors can nuance their discussion or argue it more clearly.

Reply: We thank the reviewer for their helpful observation regarding the comparison between Catalonia and Hong Kong. Our intention was not to suggest a direct sociopolitical parallel between the two contexts, but rather to illustrate a broader pattern in multilingual societies: cosmopolitan languages are often perceived as more competent or prestigious, while local languages are more strongly associated with warmth or solidarity. To clarify this, we have revised the relevant paragraph to explicitly state that the Catalonia case is presented as an illustrative example of this wider phenomenon, and not as a one-to-one comparison on lines 153 - 159

- “This discrepancy can be explained by the ideology of equality in the European Union and cultural differences […]” l. 140-141: please provide some explanation of what is meant here.

Reply: We appreciate the reviewer’s request for clarification on this point. In the revised manuscript, we have expanded and refined the explanation of this discrepancy. Specifically, we now refer to findings from Cuddy et al. (2009), which report that in some European contexts—such as Spain and Portugal—ingroup groups are rated higher on warmth but not necessarily on competence, suggesting a culturally moderated form of in-group favoritism. We also explain that in collectivist cultures like those in East Asia, individuals tend to exhibit less reference-group favoritism overall. These cultural dynamics may contribute to the lack of idealized evaluations of in-group speakers in our Hong Kong sample. We hope this elaboration addresses the concern and clarifies how cultural variation may influence stereotype content across different settings. The changes are made on lines 161-165

- The paragraph between lines 139-152 is hard to follow as it is incoherent due to information that is left implicit. It is for instance unclear what “collectivist cultures” refers to (I assume it refers to the Hong Kong case study) and what “these three languages” refers to (again, presumably the languages under scrutiny, but they have not been mentioned for a while in the text, hence the reference becomes unclear).

Reply: We thank the reviewer for pointing out the ambiguity in this section. To improve clarity, we have revised the paragraph to explicitly name the three languages under investigation (Cantonese, English, and Mandarin) and clarified that Hong Kong is the multilingual setting of focus. Additionally, we replaced the phrase “conveying a different psychological cognition” with clearer wording that better reflects the intended meaning. This section has been restructured for clarity on lines 160-171

- L. 151 mentions “implicit judgements”, but the current paper does not measure such judgements and the MGT is not an implicit attitude measure.

Reply: We thank the reviewer for this important observation. We have clarified in the manuscript that while the Matched-Guise Technique does not measure implicit attitudes in the cognitive psychology sense, it is widely interpreted in sociolinguistics and social psychology as a method for eliciting implicit social evaluations, where “implicit” refers to biases not consciously reported, not unconscious associations. This clarification distinguishes our use of the term *implicit* as referring to indirectly expressed language attitudes, rather than unconscious associations, this clarification has been added on lines 187-192

- L. 153-155: “Our current study aims to illuminate how language influences social cognition and interactions, with the overarching objective of informing inclusive policies that foster social cohesion and address linguistic biases.”

o Interactions with what?

o Throughout the text, the use of “social cognition” remains vague.

o If the aim is to inform inclusive policies, this topic should be addressed in the introduction. What sort of policies are the authors talking about? Hiring policies? Language policies? Is it even possible to address linguistic biases (again, the literature about countering biases is not addressed at all in the introduction).

Reply: We thank the reviewer for raising these important points. In response, we have revised the introduction to clarify that “interactions” refers to everyday social and professional contexts in which language use shapes listener judgments, such as hiring, service encounters, and workplace communication. We also now define “social cognition” more precisely on lines 69-71.

Regarding the mention of inclusive policy, restructured much of that paragraph to avoid overstating our claims, acknowledging that while our findings may be relevant to discussions on linguistic bias in hiring or education, further research is needed before informing concrete policy directions. These changes were made in Lines 172–183 of the revised manuscript.

- L. 163: please elaborate on what is meant by “polarized perceptions”

Reply: Thank you for your helpful comment. In the revised manuscript, we have clarified that “polarized perceptions” refers specifically to the socio-political sensitivity surrounding Mandarin in the Hong Kong context. Due to increasing political tension with mainland China—especially following the 2019 protests—Mandarin may evoke sharply divergent responses, with some listeners perceiving it as a symbol of external control and others as a pragmatic language of mobility. We have now made this explanation explicit in the manuscript to enhance clarity on 203-206.

>>Materials and methods

- How was the study framed to participants? What was the (supposed) research objective announced to them? Were participants instructed to pay attention to the content of the sentences they heard? L. 221 “[…] allowing participants to focus solely on the language of the spoken sentences.” suggests that participants may have been instructed to pay attention to language. On the other hand, if I understand correctly, language was manipulated between subject, so there would not have been language differences in the stimulus set a participant was exposed to? Please clarify.

Reply: We appreciate this insightful comment. We have revised the manuscript to clarify that participants were not informed of the study’s purpose or asked to attend specifically to language. The instruction simply asked them to listen to all recordings and confirm playback. Language was manipulated between-subjects, so each participant heard a single speaker using one language. The revisions to the manuscript are on lines 384-392.

- What background information about the participants was collected? What constituted a “Hong Kong local”? How was this operationalized?

Reply: We thank the reviewer for this question. Participants were asked to self-report their origin by selecting from “Hong Kong,” “Mainland,” or “Other.” Those who selected “Hong Kong” were retained for analysis. We agree that identity is complex and contextually shaped, but for the purposes of this study, self-identification was deemed an appropriate measure of local affiliation, particularly in the Hong Kong context. We opted not to include further probing questions (e.g., length of residence or schooling history) to avoid discomfort or reduced response accuracy. We have added a sentence in the Methods section clarifying how “Hong Kong local” was operationalized on lines 236-240.

- What were the time limits used to exclude fast/slow participants based on?

Reply: We appreciate this helpful suggestion. Participants were excluded if their total survey time was under 3 minutes or over 30 minutes, which we determined to be implausibly short or long based on internal pilot testing and consultation. We have elaborated this point on lines 241-245

- The structure of this section needs to be revisited: the participants section for instance contains information about the materials as well. Please also include separate sections for the instrument, design and procedure.

Reply: Thank you for this helpful structural suggestion. In the revised manuscript, we have reorganized the Methods section into clear subsections: Participants, Materials, Design, and Procedure.

- The authors claim that the sentences used are neutral in meaning. However, the two examples given in the paper (in Figure 1 and in the Participants section) do not seem neutral to me. “Let’s grab some biscuits to eat later” may be suggestive of the speaker’s personality and is perhaps also less compatible with a professional context (which is one of the evaluative dimensions considered in the study). Similarly, “Today, the sunshine is good” is a highly positive statement which again may reflect on how the speaker is perceived. As I have no access to the OSF materials, I cannot judge the rest of the sentences and to what extent the larger set of materials is varied and may compensate for the lack of neutrality of some sentences. I would like to ask the authors to discuss variability in ratings of the different sentences to determine to what extent their content may impact on the speaker perceptions, as the between subject design likely also steers the participant towards a focus on the content of the sentences.

Reply: We appreciate the reviewer’s concerns about sentence neutrality. While we agree that individual utterances l may carry light social or emotional tone, our goal was to reflect naturalistic, conversational speech typical of everyday Hong Kong interactions. Linguistic neutrality in naturalistic contexts is always relative; perfect affective neutrality is difficult to achieve without sacrificing ecological validity. While we cannot completely eliminate all contextual tone/flavour, we ensure that any such tone was mild, non-systematic, and evenly distributed across conditions.

To mitigate potential content-based bias, we compiled a large pool of 67 short sentences, each translated and recorded across three languages by ten fluent trilingual Hong Kong speakers, resulting in a total of 2,010 unique audio recordings, which has now been made accessible in our OSF repository. Each participant was randomly assigned to hear 10 different sentences, spoken by a single speaker in one language. This randomized structure ensured that no particular sentence or tone was uniquely associated with any one language, and that any sentence-level variation was statistically diluted and evenly distributed across the sample.

Finally, we note that while our between-subjects design may increase sensitivity to sentence content, it also prevents participants from making direct language comparisons. Combined with our use of multiple speakers and randomized sentence sets, we believe this design reduces the likelihood that sentence content alone would systematically influence trait evaluations across language groups.

- Were the voices pretested on aspects like clarity, nasality, pleasantness, etc.?

Reply: pleasantness. However, all recordings underwent a rigorous and standardized audio processing protocol, including denoising, filtering, and normalization, to ensure clarity and minimize acoustic confounds as mentioned. Additionally, pleasantness was one of the rated dimensions during the main experiment. No significant language or gender effects were observed on this trait, suggesting that vocal pleasantness did not systematically bias the results.

- How many times did the participants listen to the sentences? Just once? Please add this information to the description of the procedure.

Reply: Participants were asked to listen to each recording once and check a confirmation box before continuing. However, they could replay the recordings at any time during the rating task if desired. We have clarified this in the revised procedure section on lines 383-385 and 391-392

- Is it correct that the order of presentation of the sentences were randomized for each participant? Please make this a bit clearer in the text.

Reply: Yes, both the selection and presentation order of sentences were randomized for each participant to reduce order effects and ensure variability across responses. This has been clarified in the revised manuscript on lines 387-388

- Is the list of traits on lines 285-302 a complete list? The text mentions that it’s an “illustration”, but it’s unclear what is exactly meant by that. If this is not an exhaustive overview, please include a complete list of traits included in the questionnaire in the paper. If the list is too long for the running text, include it as an appendix. This is information is too crucial for readers to have to look for it in a separate repository. Also, please indicate which items came from which source.

Reply: Thank you for pointing this out. The list presented in the manuscript is indeed the complete set of items used in the questionnaire. To avoid confusion, we have revised the wording to clarify that this is the full list, rather than an illustrative sample. We have also added a sentence attributing the source of the items, noting that those assessing competence, trustworthiness, and goodwill were adapted from McCroskey and Teven’s (1999) Source Credibility Scale, while additional items such as attractiveness and prestige were informed by prior matched-guise studies (Chen & Cao, 2013; Coupland & Bishop, 2007; Kircher, 2015). The revisions were made on lines 355-362.

- Relatedly, based on the current description in the text, the link between the paper’s objectives of studying competence and social status and the instrument is not sufficiently clear. Why include these traits in the questionnaire? How do they link up with the constructs discussed in the introduction? What were the hypothesized underlying evaluative dimensions for these traits?

Reply: Thank you for highlighting the need to clarify the connection between the study’s theoretical objectives and the selected traits. In the revised manuscript, we now explicitly connect the measured dimensions—such as competence, prestige, and employability—to the theoretical frameworks introduced earlier. Competence and warmth map onto the Stereotype Content Model (Fiske et al., 2002), while prestige and employability reflect social status and instrumental value, consistent with Ethnolinguistic Vitality Theory (Giles et al., 1977) and prior Hong Kong sociolinguistic literature (Lai, 2007; Evans, 2011). We have also clarified that these traits were selected to capture both interpersonal and pragmatic evaluations relevant to language-based social judgments. Revisions were made on lines 319-326

- What did the definitions in the questionnaire look like?

Reply: We thank the reviewer for this query. Definitions for each trait were provided beneath the rating scale to ensure participant clarity (e.g., “Considerate: shows concern for others’ feelings and needs”). These were presented in plain language and based on the constructs’ intended interpretation in their respective source measures. We will upload a sample of the questionnaire (including definitions and visual format) to the OSF repository.

- What type of scales where used? In Figure 1B I can see indications of a seven point semantic differential, but Figure 1C has both 7 point and 5 point ratings on the y-axis. Why use different types of scales?

Reply: We acknowledge the reviewer’s observation regarding scale variation. The questionnaire drew items from different prior studies, some of which used 5-point scales (e.g., Coupland & Bishop, 2007) while others used 7-point semantic differentials (e.g., McCroskey & Teven, 1999). We retained the original response formats to preserve the psychometric integrity and comparability of each construct. Nonetheless, we agree that this design choice could have been more clearly explained and have clarified this in the manuscript on lines 326-329.

- L. 270-274: please refer to previous work on the link between language attitudes and employability to justify and explain the relevance of your choices (e.g. Hosoda & Stone-Romero 2010). This topic should also be prepared more extensively in the introduction.

Reply: We appreciate the reviewer’s suggestion to better contextualize the employability measures. We have now included references to prior work linking language attitudes with perceptions of employability on lines 330-337

- Lines 300-302 contain questions regarding employment including a hierarchical perspective. This is an interesting approach, but please prepare this aspect of the study in the introduction which so far only discusses employability which pertains to question 3 only. What is the motivation for also including questions 2 and 4 and what are the hypotheses regarding these questions?

Reply: We thank the reviewer for highlighting the need to clarify the motivation behind the inclusion of employment-related items. While our initial focus was on general employability (i.e., openness to hiring the speaker), we expanded the set of questions to capture a broader range of social role evaluations that vary in hierarchy and relational framing. Specifically, items such as being employed *by* the speaker or working *with* the speaker were designed to assess how language-based perceptions influence willingness to engage in upward, lateral, and downward professional relationships. This decision was inspired by the idea that language attitudes may affect not just hiring preferences, but broader collaboration dynamics in multilingual environments (cf. Hosoda & Stone-Romero, 2010). Although these items were exploratory and not guided by strong a priori hypotheses, we expected higher ratings for English speakers in these dimensions due to English’s association with professional competence and global status in Hong Kong.

- Items F and G on lines 291-292 both contain “sensitive”. Is this a typo?

Thank you for catching this. Yes, this was a typographical error. The duplicate item has now been corrected in the revised manuscript, now on line 369.

- It is unclear to me whether it was disclosed to participants that they heard one speaker and they rated the speaker of the ten sentences once after hearing all 10 or whether they rated the speaker after each sentence (as is customary in more traditional within-subject MGT designs). I assume it’s the former, but given that most MGT studies work within-subject, it would be good to make this more explicit in the description of the design.

Reply:	We appreciate this comment. In this between-subjects design, each participant listened to ten sentences spoken by the same speaker in one language. They were asked to rate the speaker **once**, after hearing all ten recordings. This was done to preserve the illusion of a single, naturalistic interaction and to avoid drawing attention to the manipulation. We have clarified this more explicitly in the Procedure section of the revised manuscript on lines 384-386

>>Results

- How many ratings per sentence (in each of the languages) were collected?

Reply: We appreciate the reviewer’s question. Each participant was randomly assigned to one of 30 speaker-language conditions (10 speakers × 3 languages), and within each condition, they were exposed to 10 out of 67 possible sentences, all spoken by the same speaker in the same language. The sentence selection was randomized, and the Qualtrics platform was configured to evenly present the sentence pool across participants while preserving randomization. As a result, the number of ratings per sentence was approximately equal within each language condition, though not strictly uniform due to random assignment as well as dropout and exclusions. Given the large number of total participants (N = 541) and 67 sentences per language, each sentence received an estimated 25–30 ratings per language condition. This has been clarified one lines 405-406.

- What was the impact of individual speakers and individual sentences on the ratings?

Reply: We thank the reviewer for raising this point. We acknowledge that the current study design was not optimized for sentence-level analysis. With 67 sentences distributed across 544 participants (each of whom rated 10 different sentences), the number of observations per sentence was insufficient to support reliable statistical comparisons.

Regarding speaker-level effects, we conducted exploratory one-way ANOVAs on select evaluative dimensions and found some statistically significant differences across the 10 speakers (e.g., F(9,211) = 2.83, p = .004 for competence). However, given the between-subjects design and the sample size per speaker group (~50–60 participants), these results should be interpreted cautiously. We have added a brief note about speaker effects in the Results section to reflect this exploratory analysis.

- The current analysis does not follow the standards of the field. Matched guise data of the type presented in this study are first submitted to a factor analysis (or alternatively a principal component analysis) for dimension reduction to detect the underlying structure of the evaluations. Next, I would recommend the authors to conduct a mixed effects regression analysis per evaluative dimension taking into account speaker and sentence in the random effects structure to account for their impact on the results.

Reply: We thank the reviewer for this helpful suggestion. While factor analysis is sometimes used for dimension reduction in matched-guise studies, our approach was both theory- and scale-driven. Traits were selected based on their alignment with the Stereotype Content Model, and were measured using items adapted from McCroskey and Teven’s (1999) validated Source Credibility Measure. As these scales were developed through prior factor analysis and have demonstrated high reliability, we did not conduct further CFA or PCA. Internal consistency was high: Trustworthiness (α = .87), Competence (α = .88), and Goodwill/Benevolence (α = .87); these are now reported on lines 420–425.

Additionally, many published MGT studies have used trait means without dimensionality reduction, particularly when using validated or theory-based constructs (e.g., Lieberman, 1975; Ioratim-Uba, 2001; Chen & Cao, 2013; O’Rourke & Ramallo, 2013).

Regarding mixed-effects regression, our design confounded language and speaker identity, and participants heard 10 randomly selected sentences from a pool of 2,010 (67 sentences × 3 languages × 10 speakers). This randomization was intended to distribute speaker and sentence variance across conditions, making their influence function more like random noise than a structured confound. We therefore opted for between-subjects ANOVA, consistent with prior MGT studies using similar designs.

>>Discussion

- The discussion does not relate the results to the wider language attitudes research tradition and as such does not explicitly contribute to theorizing in the field at large beyond the current case study (as interesting a case as that may be).

Reply: We thank the reviewer for this valuable observation. In response, we have added a new paragraph to the Discussion section that explicitly situates our findings within the broader language attitudes literature and theoretical frameworks such as the Stereotype Content Model and Ethnolinguistic Vitality Theory. In particular, we highlight how the observed trait evaluations reflect global patterns—such as the association of high-status languages with competence and local varieties with solidarity—while also revealing the unique sociopolitical dynamics shaping language attitudes in postcolonial Hong Kong. This addition clarifies how our case study extends and nuances existing theorizing in the field. The changes are made on lines 598-609

>>Limitations and future directions

- I feel that the point on the varieties of English and Mandarin included in the study is not necessarily a limitation. Given the purpose of the study to look at language attitudes of Hong Kong locals towards the three main local languages, it makes sense to include Cantonese-accented varieties of English and Mandarin in the study. However, it is crucial to include this information in the description of the methodology and make clear in the introduction that the perspective is on the local varieties of English and Mandarin. The possibility of looking at attitudes towards other varieties of English and Mandarin could then still be mentioned in this section as a suggestion for follow-up research.

Reply: We appreciate the reviewer’s point and agree that it is important to clarify the nature of the speech samples used in our study. Our goal was to examine perceptions of language use as encountered in real-world Hong Kong contexts, and therefore we did not attempt to standardize speaker accent or proficiency across languages.

The speakers were all Hong Kong-born trilinguals; however, they differed in language backgrounds, with some having studied or lived abroad. Thus, while some recordings may exhibit accent features associated with Hong Kong, we cannot make definitive claims about whether they conform to "Hong Kong English" or "Hong Kong Mandarin" norms, nor was this our intention. We have revised the Methodology and Introduction to clarify on lines 266-269 that speaker variability was not experimentally manipulated. We also revised the Limitations section to acknowledge this constraint and suggest that future studies might explore the influence of accent more directly, perhaps by using controlled stimuli with listener-rated accent strength. The revision is added on lines 631-634.

- This section is very limited.

Reply: Thank you for pointing this out. We agree that the section was overly brief and have now expanded it to provide a more detailed reflection on the methodological implications and future directions regarding accent, speaker variation, and measurement limitations.

>>Minor comments:

- l. 92-93: “Like many multilingual societies, different languages are 93 associated with specific settings” > like in many …

- l. 112-113, l.118-121: please revisit the structure of this sentence

- l. 195 “provided with informed written consent” > provided written informed consent

- l. 225: what is meant by “identities” here?

- L. 256: “to” missing

Reply: We thank the reviewer for their attentive reading and helpful suggestions. All minor editorial issues have been addressed as follows:

Line 92: Corrected “the deeply rooted stereotypes that underlies it” to “underlie” to maintain subject-verb agreement.

Line 95: Removed the extra space in “language use ” to read correctly as “language use.”

Lines 112–113: Revised the sentence for grammatical consistency and parallel structure to:

“This reflects a stronger emotional identification with their mother tongue and demonstrates how the local language evokes sentiments of in-group membership.”

Line 264: Corrected “the items […] assesses” to “the items assess” for verb agreement.

These corrections have been implemented on lines 104; 108; 135–137; and 349 of the revised manuscript.

### Reviewer #3:

### The paper investigates attitudes toward speakers of Cantonese, English, and Mandarin in Hong Kong using the matched-guise technique, a well-established method in sociolinguistic research.While traditional matched-guise studies typically employ a within-subjects design, the authors opt for a between-groups approach in this study. This methodological choice is justified as a way to minimize participants' awareness of the experimental manipulation and to avoid recognition of the same speakers across conditions. Random assignment to one of the three language conditions helps reduce the risk of systematic bias due to group differences. However, the paper would benefit from more detailed descriptive statistics on participant demographics. Reporting at least age and gender distribution for each language group would enhance confidence that the groups are indeed comparable and that any observed differences in attitudes are attributable to language rather than underlying demographic variation.

Reply: We appreciate the reviewer’s recognition of our methodological rationale.

While the study employs 10 speakers (5 male and 5 female), each participant is exposed to only one speaker in one language. The paper does not clarify whether each language condition included a balanced representation of speakers (by gender or other characteristics). The authors mentioned that they control for variables such as gender and identities across participants – however, this should be explained in more detail.

Reply: We thank the reviewer for pointing out the ambiguity in our description. While we did not explicitly state that we “controlled for variables such as gender and identities,” our original phrasing — “ensuring variation in speaker gender and identities across participants” — may have unintentionally suggested this. To clarify we ensured a balanced and randomized assignment of participants across 30 total conditions (10 speakers × 3 languages), such that each participant was randomly assigned to hear one speaker in one language, with speaker gender and identity evenly distributed across the three language conditions. In the revised manuscript, we will revise this sentence to better reflect that speaker—not participant—characteristics were controlled in the assignment procedure and explain the randomization mechanism in more detail. The revision is made on lines 289-290.

The authors appropriately acknowledge a potential confound in the study: the use of Mandarin speakers with Hong Kong accents. Although not mentioned, it can be assumed that the English guises were also accented. As a result, the manipulation blends language and accent, which complicates the interpretation of findings. This limitation is important, as listeners' attitudes may be shaped by both linguistic variety and perceived social identity indexed by accent. It is worth considering if the paper should be reframed as evaluations of accents and not languages. The studies conducted by Woolard (1984, 1989, 2009) and later by Newman et al. (2008) in Catalonia show how participants may react to members of their in-group using other languages. Nejjari et al. (2019) bring relevant information about native and non-native accents.

Reply: While our study used a between-subjects design rather than a traditional within-subjects matched-guise test, the use of naturally fluent trilingual speakers introduces the common challenge of balancing naturalness and experimental control. Complete elimination of accent is difficult without compromising ecological validity, particularly in sociolinguistic contexts where accents carry meaningful identity cues. In Hong Kong, English spoken with a local accent is widespread and socially embedded, especially among younger and university-educated populations. As such, the use of locally accented English may reflect typical usage and contribute meaningfully to understanding language attitudes in the city’s sociocultural context. While this may introduce some degree of confound, it also reflects real-world language usage and is thus relevant for capturing social perception in context. In contrast, accented Mandarin carries greater sociopolitical salience due to ongoing tensions between Hong Kong and the Mainland. Previous research suggests that Mandarin spoken with a local accent may be interpreted through the lens of local–Mainland identity dynamics. Nevertheless, we acknowledge that we did not obtain listener-rated accent strength, and we have clarified in the manuscript that future work should more directly assess how accent interacts with social perception across language conditions.

Regarding the statistical analyses, more details should have been provided. It can be inferred that Welch's was used because the assumption of homogeneity of variances was violated. No information is provided regarding the normality assumptions, to know if Kruskal-Wallis had been necessary.

Reply: While the assumption of homogeneity of variances was met for most traits, we used Welch’s ANOVA throughout the analysis for consistency and robustness. As our sample size is large and reasonably balanced, Welch’s test yields results comparable to standard one-way ANOVA but with a slightly more conservative threshold, which does not affect the interpretation of our findings. In addition, that the assumption of homogeneity of variances was violated across all key traits (p < .05). As such, Welch’s ANOVA is the more appropriate analytical choice, and we have clarified this justification in the revised manuscript on lines 422-428

Overall, the paper is well-written and clearly articulated. However, there are a few areas where the text would benefit from revision to enhance grammatical accuracy and clarity. For example:

• Line 92: "the deeply rooted stereotypes that underlies it" should be corrected to "underlie" to maintain subject-verb agreement.

• Line 95: "This division in language use provides" contains an extra space and should be revised to "language use."

• Lines 112–113: The sentence "Reflecting a stronger emotional identification with their mother Tongue and demonstrates how the local language evokes sentiments of in-group membership" is grammatically inconsistent. Consider revising for parallel structure, e.g., "This reflects a stronger emotional identification with their mother tongue and demonstrates how the local language evokes sentiments of in-group membership."

• Line 264: The phrase "the items […] assesses" contains an incorrect verb agreement. It should be revised to "the items assess."

Reply: We thank the reviewer for their attentive reading and helpful suggestions. All minor editorial issues have been addressed as follows:

Line 92: Corrected “the deeply rooted stereotypes that underlies it” to “underlie” to maintain subject-verb agreement.

Line 95: Removed the extra space in “language use ” to read correctly as “language use.”

Lines 112–113: Revised the sentence for grammatical consistency and parallel structure to:

“This reflects a stronger emotional identification with their mother tongue and demonstrates how the local language evokes sentiments of in-group membership.”

Line 264: Corrected “the items […] assesses” to “the items assess” for verb agreement.

These changes can be found on lines 104; 108; 135–137; and 349 of the revised manuscript.

The authors state that all data are fully available; however, the provided link was not accessible, as it required permission to view the files.

Reply: Thank you for bringing this to our attention. The OSF link originally included in our submission was temporarily flagged due to an internal spam detection issue on the OSF platform. We have contacted OSF support, and the issue has now been resolved. The data repository is once again fully accessible to the public.
