# Supplementary material for: Spoken language and attitudes in Hong Kong: English leads in prestige, mandarin rises in employability, and Cantonese faces challenges
Source: PLoS One. 2026 Mar 9;21(3):e0343611. doi: 10.1371/journal.pone.0343611 (PMC12970906; doi:10.1371/journal.pone.0343611)
Supplement: Attachment — Submitted filename: Reply to Reviewer 21 11 2025.docx [file pone.0343611.s004.docx]

Dear Editors,

Thank you for the opportunity to revise our manuscript. We have carefully addressed all comments from the editor and reviewers.

Major revisions include removing the Significance Statement, restructuring the *Materials and Methods* section to eliminate repetition, relocating the Data Availability statement to the metadata, and adding full details on speaker characteristics (L1/L2 status and age of acquisition).

We clarified the recruitment and instruction languages, reorganized several methodological descriptions, and removed procedure-related content from *Instruments*.

Following the editor’s summary, we also added a brief paragraph in the Limitations section acknowledging the trade-off inherent in having each listener rate only one speaker in one language (Lines 629–632).

Figures were repositioned according to first mention, and the full manuscript underwent a further round of proofreading to correct minor issues.

Reviewer-specific concerns—including argument completion, conceptual clarifications, translation/back-translation procedures, and definitions of key terms—have all been addressed in the revised text.

We believe the manuscript is now substantially clearer and fully responsive to the requested revisions.

Best regards,

Xiangbin TENG

**Reply to editor’s comment**

- Remove the significance statement; the journal does not require one.

  **Reply: Thank you for bringing this to our attention. The section has been removed.**
- The structure of the 'Materials and Methods' section should be improved:
  - Remove the text directly after 'Materials and Methods' heading, as this text is repeated elsewhere in the section.

**Reply: Thank you for the comment, the repeating text has been removed from the Materials and Methods sections**

- The data availability statement can be provided in the article metadata rather than in the main text.

**Reply: Thank you for the comment, the text has been removed and added to the metadata**

- Remove information regarding procedure from under 'Instruments'.

**Reply: Thank you for the comment, the text has been removed**

- Check for repetition, for example lines 247-252, as pointed out by Reviewer 4.

**Reply: Thank you for the comment, the repeating text has been removed**

- Provide more detailed information about speaker characteristics under Instruments, particularly the fact that they were L2 speakers of some of the languages tested, as per Reviewer 5's comment.

**Reply: Thank you for the comment, we have added a detailed description of speaker characteristics in the Instruments section, specifying which languages were acquired as L1 versus L2 and the approximate age of acquisition for later-learned languages. The new text is on lines 279-290.**

- In the limitations, discuss the procedural choice to have each listener rate one speaker in one language, as per Reviewer 4's suggestion.

**Reply: Thank you for your reply, we intentionally had each listener rate only a single speaker to drastically reduce the time required to complete the questionnaire, thereby maximizing participation. While this approach collects less data per participant, it allows for a larger overall sample, a trade-off between depth and breadth. Because this was an intentional methodological choice rather than an oversight, we do not consider it a limitation needing explicit mention.**

**Nevertheless, we understand the concern here and readers could conduct further studies to enrich the findings here. We added to line 629 – 632.** *‘Because each listener rated only one speaker in a single language, this design reduced participant burden and allowed a large sample, but limited within-listener comparisons. Future work using multiple speakers per listener would enable stronger cross-speaker controls.’*

- Figures should be placed after they are first referred to in the text.

**Reply: Thank your suggestion. Figure 1 already appears immediately after its first mention, at the end of the Introduction, and appropriately bridges to the Methods section. We have therefore retained its position. Figure 2 has been moved to the results section at the end of first mention**

- Please do another round of proofreading of the article.

**Reply: Thank you for bringing this to our attention, we have done another round of proofreading for the manuscript.**

**Reply to Reviewer 4’s comment**

Reviewer #4: The manuscript entitled "Spoken Language and Attitudes in Hong Kong: English Leads in Prestige, Mandarin Rises in Employability, and Cantonese Faces Challenges" investigates Hong Kong listeners’ language attitudes towards English, Mandarin and Cantonese in a matched guise experiment. It finds that English is rated higher on prestige measures, and Mandarin on employability. The research is timely, well-executed and will be of interest to the journal’s readership. I have some minor suggestions below.
Sometimes the structure is inefficient. In terms of positioning of figures, it is unhelpful that Fig 1 is at the end of lit review and Fig 2 at end of method. They should be positioned straight after the text that references them.

**Reply: Thank your suggestion. Figure 1 already appears immediately after its first mention, at the end of the Introduction, and appropriately bridges to the Methods section. We have therefore retained its position. Figure 2 has been moved to the results section at the end of first mention**

The structure could be made more efficient. In particular, in method repetitive information is presented in different places. E.g. some information under Materials & Methods and Participants is repetitive. L.247-252 – repetitive sentences

**Reply: Thank you for the comment, the repeating text has been removed from the Materials and Methods sections**

I am also not used to having the data and code availability section within the main text like that.

**Reply: Thank you for the comment, the text has been removed and added to the metadata**

Methodologically, it is important to report in what language the participants were recruited and instructed, as this might affect their perception of the guises.

**Reply: We thank the reviewer for highlighting this important point, the questionnaire and its instruction is in English, which has been uploaded to our OSF folder, while the recruitment message that we sent out are in both English and Chinese, in formal contexts like as that of the recruitment message, written Chinese is semantically the same for both Mandarin and Cantonese, this information has been added to the manuscript on lines 228-230**

My understanding of the method is that each listener provided a single suite of ratings on the different scales for one speaker in one language. This sounds like an inefficient data collection method. It would have been better to have each listener listen to several speakers. That would also strengthen the stats as it would allow for comparability across listeners. This flaw does not discount the paper’s contribution but should be acknowledged in the limitations.

**Reply: Thank you for your reply, we intentionally had each listener rate only a single speaker to drastically reduce the time required to complete the questionnaire, thereby maximizing participation. While this approach collects less data per participant, it allows for a larger overall sample, a trade-off between depth and breadth. Because this was an intentional methodological choice rather than an oversight, we do not consider it a limitation needing explicit mention.**

**Nevertheless, we understand the concern here and readers could conduct further studies to enrich the findings here. We added to line 629 – 632.** *‘Because each listener rated only one speaker in a single language, this design reduced participant burden and allowed a large sample, but limited within-listener comparisons. Future work using multiple speakers per listener would enable stronger cross-speaker controls.’*

Minor
There are multiple typos throughout the manuscript. Here are the 1st four, but there are more in the later parts of the document.
L112: People evaluates -> evaluate
L119: too socially categorise -> to socially categorise
L172: insights how language -> insights into how language
L221: recruitment starts from -> recruitment lasted

**Reply: Thank you for bringing this to our attention, we have done another round of proofreading for the manuscript.**

**Reply to Reviewer 5’s comment**

Reviewer #5: Thank you for your manuscript! This is a clear, well-written manuscript on a relevant language attitudes but also World Englishes topic, and I recommend a minor review. The abstract offers a good overview of the study and its main conclusions. I do not see the relevance of the significance statement, since it is virtually the same as the abstract. The general set up, research gap and theoretical foundation of the study are described clearly and concisely. The method section does require some more work, but the results section provides a sufficient overview of the analyses and main patterns observed. The discussion and conclusion are well-written and clearly repeat the main contributions of this paper. Please see specific comments below.

Line 93: This makes… I do not agree that the logical conclusion to the features of Hong Kong listed make this an ideal site for studying how social cognition can be influenced by language perception and underlying stereotypes. Can you complete the argument? Is it its dynamism, current political changes, general diversity, or all combined? If you offer a main statement that includes all of the mentioned factors that will clarify your statement better.

**Reply: Thank you for your suggestion: we added a main statement that clarified the argument on lines 87-90**

Line 127: ‘Likewise’ needs to be replaced with a better linker, but preferably explained in the sense that the first example in the paragraph illustrates a relatively universal phenomenon and so not something that is unique to Asia.

**Reply: Thank you for your suggestion, the linker has been replaced. Lines 124-125**

Line 131: what is structural vitality of a language? Can you add a brief explanation?

**Reply: Thank you for your comment, a brief explanation has been added. On line 130**

Line 158: ‘model’ refers to what?

**Reply: Thank you for brining this to our attention, the word model has been replaced with explicit naming of SCM on line 154**

Line 205: the matched-guise technique merely refers to the fact that one speaker produces all varieties tested, not that this has to take place in a within-subjects design. Please also refer to your study as having a between-subjects design, which I believe it does.

**Reply: Thank you for this comment. The section has since been substantially reorganized, and the sentence in Line 205 no longer appears in the revised manuscript. We have clarified that the study uses a between-subjects design on line 311.**

Lines 219-240. Remove the repetition of information. Introduce the method section generally and offer specific details in the sections.
**Reply:Thank you for bringing this to our attention, the section has been reworked**

Line 242: ‘Hong Kong local identity’ is too vague. Not born and raised in Hong Kong? Expats and how long have they lived in Hong Kong?

**Thank you for pointing out the need for clarification. In this study, Hong Kong local identity was operationalised entirely through self-identification, as participants selected “Hong Kong” in the identity question. This approach aligns with sociolinguistic research in Hong Kong, where self-reported identity reliably reflects perceived group membership. We have now made this explicit in the Participants section lines 236-239**

Line 255: many women participated, potential impact on responses versus men?

**Reply: Thank you for your comment we have added acknowledgment of gender imbalance and justification on lines 250-251**

Lines 264-280: how did you check the translations? What method did you employ?

**Reply: We added a description of the translation and back-translation process on lines 256-257**

Line 287: Why add information on the procedure when explaining stimuli development? That is confusing.

**Reply: Thank you for brining this to our attention. The procedure-related text has been removed from Instruments.**

Line 625: It does not seem sincere to not have been clearer from the beginning (method chapter) about the language skills of the selected speakers. This immediately raises doubt in terms of you pre-testing the stimuli rigorously enough to be sure that each fragment was representative of the languages under study. Please communicate the characteristics of each speaker and their language skills in more detail in the method.

**Reply: Thank you for your suggestion, detailed speaker background information, including L1 status, age of acquisition, and fluency, has been added to the Instruments section lines 279-290**
