# Supplementary material for: Spoken language and attitudes in Hong Kong: English leads in prestige, mandarin rises in employability, and Cantonese faces challenges
Source: PLoS One. 2026 Mar 9;21(3):e0343611. doi: 10.1371/journal.pone.0343611 (PMC12970906; doi:10.1371/journal.pone.0343611)
Supplement: Attachment — Submitted filename: Response letter.docx [file pone.0343611.s005.docx]

Dear Dr. Berghoff,

Thank you for your detailed guidance on our revision. We have carefully revised the manuscript to address all points raised, and the full paper has additionally undergone comprehensive professional editing for language, grammar, consistency, and flow. Below we respond point-by-point to the specific items you flagged, with the corresponding locations (line numbers) in the revised manuscript.

**Language errors**

- **Line 119 (“which its…”)** → corrected to “including its …” (revised manuscript, **line 119**).
- **Line 122 (“would serves…”)** → corrected to “would serve …” (subject–verb agreement; revised manuscript, **line 122**).
- **Hyphenation inconsistency (“competence related”; “ingroup”)** → standardized terminology throughout (e.g., “competence-related,” “in-group/out-group”) for consistent academic usage (changes applied across the manuscript).
- **Line 145 (missing period after “et al”)** → corrected to “et al.” (revised manuscript, **line 151**).
- **Line 160 (“multilingual society like Hong Kong”)** → wording revised for clarity and grammatical correctness (revised manuscript, **line 51**).
- **Line 246 (“These sentences For example”)** → rewritten for grammatical correctness and readability (revised manuscript, **line 237**).
- **Lines 221–222 (run-on sentence)** → revised for clarity and sentence structure (changes implemented in the Participants section).

In addition to the items above, we conducted a comprehensive review of the full manuscript and corrected additional language issues throughout (grammar, punctuation, tense consistency, spelling/usage consistency, and awkward phrasing).

**Structure and organization**

- **Lines 166–174 (method-specific content should not be in Introduction)** → removed from the Introduction and incorporated into the appropriate Methods subsections (revised manuscript, **lines 340–347**).
- **Lines 329–333 (recruitment information)** → reorganized so that recruitment details are clearly presented in the Participants section; redundant text was removed to improve flow.
- **Line 329 (Qualtrics usage should be in Procedure)** → moved to the Procedure subsection (revised manuscript, **line 357**).
- **Lines 335–358 (questionnaire/scales should be in Instruments)** → consolidated questionnaire/scales content into the Instruments section, together with other measurement details.
- **Lines 481–485 (method commentary does not belong in Results)** → moved to a dedicated Statistical analysis subsection in Methods (revised manuscript, **lines 373–384**).
- **Repetition at the start of Discussion** → revised to remove redundant re-statements of the main findings and improve paragraph transitions.
- **Repeated text between lines 547–570** → revised to remove redundancy and tighten the Mandarin/accent explanation (revised manuscript, **lines 545–549**).

We believe these revisions fully address the language and structural concerns and substantially improve the clarity and readability of the manuscript. We appreciate your time and guidance and thank you again for the opportunity to revise.

Sincerely,
Xiangbin Teng
(on behalf of all authors)
