# Supplementary material for: Spoken language and attitudes in Hong Kong: English leads in prestige, mandarin rises in employability, and Cantonese faces challenges
Source: PLoS One. 2026 Mar 9;21(3):e0343611. doi: 10.1371/journal.pone.0343611 (PMC12970906; doi:10.1371/journal.pone.0343611)
Supplement: Attachment — Submitted filename: PONE-D-25-1768R1_Manuscript reviewer comments Oct 25.docx [file pone.0343611.s002.docx]

Reviewer Comments

Thank you for your manuscript! This is a clear, well-written manuscript on a relevant language attitudes but also World Englishes topic, and I recommend a minor review. The abstract offers a good overview of the study and its main conclusions. I do not see the relevance of the significance statement, since it is virtually the same as the abstract. The general set up, research gap and theoretical foundation of the study are described clearly and concisely. The method section does require some more work, but the results section provides a sufficient overview of the analyses and main patterns observed. The discussion and conclusion are well-written and clearly repeat the main contributions of this paper. Please see specific comments below.

1) Line 93: This makes… I do not agree that the logical conclusion to the features of Hong Kong listed make this an ideal site for studying how social cognition can be influenced by language perception and underlying stereotypes. Can you complete the argument? Is it its dynamism, current political changes, general diversity, or all combined? If you offer a main statement that includes all of the mentioned factors that will clarify your statement better.
2) Line 127: ‘Likewise’ needs to be replaced with a better linker, but preferably explained in the sense that the first example in the paragraph illustrates a relatively universal phenomenon and so not something that is unique to Asia.
3) Line 131: what is structural vitality of a language? Can you add a brief explanation?
4) Line 158: ‘model’ refers to what?
5) Line 205: the matched-guise technique merely refers to the fact that one speaker produces all varieties tested, not that this has to take place in a within-subjects design. Please also refer to your study as having a between-subjects design, which I believe it does.
6) Lines 219-240. Remove the repetition of information. Introduce the method section generally and offer specific details in the sections.
7) Line 242: ‘Hong Kong local identity’ is too vague. Not born and raised in Hong Kong? Expats and how long have they lived in Hong Kong?
8) Line 255: many women participated, potential impact on responses versus men?
9) Lines 264-280: how did you check the translations? What method did you employ?
10) Line 287: Why add information on the procedure when explaining stimuli development? That is confusing.
11) Line 625: It does not seem sincere to not have been clearer from the beginning (method chapter) about the language skills of the selected speakers. This immediately raises doubt in terms of you pre-testing the stimuli rigorously enough to be sure that each fragment was representative of the languages under study. Please communicate the characteristics of each speaker and their language skills in more detail in the method.
